# The polymorphic nature of the human dopamine D4 receptor gene: A comparative analysis of known variants and a novel 27 bp deletion in the promoter region

**DOI:** 10.1186/1471-2156-6-39

**Published:** 2005-06-28

**Authors:** E Szantai, R Szmola, M Sasvari-Szekely, A Guttman, Z Ronai

**Affiliations:** 1 Department of Medical Chemistry, Molecular Biology and Pathobiochemistry, Semmelweis University, Budapest, Hungary; 2 Department of Genetics, Eotvos Lorand University, Budapest, Hungary; 3 Marie Curie Chair of the EC, Horvath Lab. of Bioseparation Science, Institute of Analytical Chemistry and Radiochemistry, Leopold-Franzens University Innsbruck, Austria

## Abstract

**Background:**

The human dopamine D4 receptor (DRD4) is a candidate gene of great interest in molecular studies of human personality and psychiatric disorders. This gene is unique in having an exceptionally high amount of polymorphic sites both in the coding and in the promoter region.

**Results:**

We report the identification of a new 27 bp deletion starting 524 bp upstream of the initiation codon (27 bp del) of the dopamine D4 receptor (DRD4) gene, in the close vicinity of the -521C>T SNP. The presence of the 27 bp deletion leads to the misgenotyping of the -616C>G SNP by the *Sau*96 I RFLP method, thus the genotype determination of the mutation is of additional importance. The frequency of this novel sequence variation is considerably low (allele frequency is = 0.16%), as no homozygotes, and only 3 heterozygote carriers were found in a healthy, unrelated Caucasian sample (*N *= 955).

**Conclusion:**

Remarkably, the deleted region contains consensus sequences of binding sites for several known transcription factors, suggesting that the different alleles may affect the transcriptional regulation of the gene. A comparison of methods and results for the allelic variations of the DRD4 gene in various ethnic groups is also discussed, which has a high impact in psychiatric genetic studies.

## Background

The dopaminergic system has received a significant amount of attention due to the important role it plays in the central nervous system in motor control, cognition, reward, emotion and endocrine regulation [1]. Recently, the D4 dopamine receptor (DRD4) gene [GenBank:U95122, GenBank:L12397] has been the target of psychogenetic studies mainly because it possesses a high number of polymorphisms [2], presumably as a consequence of its subtelomeric chromosomal localization (11p15.5) [3,4]. Polymorphisms in the DRD4 gene have received particular attention in the past decade because of their possible role in mental disorders [5-8], substance abuse [9-11] and the normal variations of human personality [12-16].

The gene and its regulatory region contain several length variations and numerous single nucleotide polymorphisms (SNPs). Much interest has been focused on these sequence variants in the investigation of complex neurobehavioral disorders using association study methods.

However, results of the psychogenetic association studies are often controversial. There are several reasons for the lack of conclusive results, such as the dangers of population stratification and the small effect size of individual genes complicated by oligogenic interactions [17]. Failure to confirm associations determined by various laboratories is partially explained by differences in demographic and ethnic structure of the subsequent studies, as a consequence of the diversity in allele frequencies of several polymorphisms among different populations. Moreover, differences in phenotyping and genotyping methodology may also obscure the weak effect of a gene variant on the investigated trait.

The human dopamine D4 receptor gene, located proximal to the Harvey-RAS oncogene locus and distal to the tyrosine hydroxylase locus [18], consists of 4 exons. The third exon contains a polymorphic 48 bp repeat region, the repeat number varies between 2 and 11. This part of the gene encodes the putative third cytoplasmic loop, and thus the polymorphism changes the length of the receptor protein [19]. Given that the variable number of 48 bp repeat (VNTR) is in a region that couples to G proteins and mediates postsynaptic effects [20], association studies have generated considerable interest. The DRD4 VNTR has been widely studied since a striking association was described between the seven-repeat allele and the human personality trait of increased novelty seeking [12,13], although the replications of the initial findings have been controversial [9,21]. So far evidence suggests that there is an association between the DRD4 7 repeat allele and attention deficit hyperactivity disorder (ADHD) also, although the effect size is small [6].

Further polymorphisms of the coding region of the DRD4 gene were also identified, [5,22-24], moreover additional mutations and polymorphisms have been described just recently in the introns as well [25-27].

**Figure 4:**
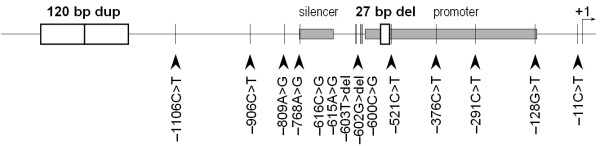
**Location of the 27 bp deletion and other polymorphic sites in the promoter region of the human DRD4 gene. **The deleted region starts 524 bp upstream of the initiation codon. Figure is drawn to scale and each position is shown relative to the first nucleotide of the initiation codon indicated as +1. A cell type-specific promoter region between -591 and -123 contains the promoter of the gene, and the negative modulator is between the -770 and -679 positions (grey boxes). Open boxes indicate length variants at the DRD4 promoter region. Arrowheads specify the positions of SNPs.

Analysis of the 5' upstream region of DRD4 gene revealed that the promoter region is probably located in the region between -591 and -123 relative to the initiation codon, with a negative modulator between the -770 and -679 positions [28]. This region of the DRD4 gene is also astonishingly abundant in polymorphisms (see Fig. 4), including numerous SNPs [29,30], and a 120 bp tandem duplication [31].

The -521C>T SNP is located on a CpG island at one end of a cell-type specific regulatory element [28]. The -521C allele was shown to be 40% more active than the T allele in an *in vitro *transient expression system [32]. Moreover, a significantly higher incidence of the CC genotype was found among schizophrenics [32]. The SNP has also been associated with novelty seeking behavior in separate studies of Japanese, Caucasian and Afro-American samples [16,33,34], but negative results were also described [35-37]. Meta-analyses of existing studies have been conducted to provide statistical measures of the small association between -521 C>T SNP genotypes and novelty seeking [38].

A regularly investigated polymorphism in the promoter sequence of the DRD4 gene is the -616C>G SNP that may result in the gain of an AP-2 transcription factor binding site [39] and thereby affects the expression characteristics of the receptor. Associations of the -616C>G SNP with ADHD [39,40], schizophrenia [41] and personality dimensions [33] were studied but did not yield consequent results. Barr et al.[39] added the -616C>G SNP to the investigations of the 5' upstream region by studying the haplotype transmissions of three polymorphisms in the promoter region and the 48 bp VNTR in exon III as genetic risk factors for ADHD. An association between ADHD and the -616C>G SNP was reinforced recently [40].

An additional length polymorphism of the 5' untranslated region of the DRD4 gene has been reported as common in the population. The polymorphism consists of a 120 bp tandem duplication 1.2 kb upstream from the initiation codon [31] and gives rise to the *Pst *I RFLP previously reported [42]. Association studies between the 120 bp duplication and the occurrence of ADHD hypothesized the potential significance of this region in the regulation of transcription [43]. Experimental evidence suggests enhanced binding capacity of Sp1 transcription element to the duplicated form [44]. The duplication is abundant in the human race, although allele frequencies do vary among different ethnic groups (see Discussion).

Identification of new, perhaps functional length variants is especially intriguing because the up-regulation of D4 receptors may be more significant than any changes in the protein structure [45,46]. In previous publications the density of DRD4 was found to be six fold elevated in the brains of schizophrenic patients [47], and the DRD4 mRNA was elevated in the frontal cortex of schizophrenics in post mortem studies in comparison with controls [47], pointing to possible allelic variants that influence the transcription levels of the DRD4 gene. The -521C>T SNP has been shown to affect transcriptional activity in an *in vitro *transient expression system [32]. In the present study we characterized a new deletion mutation in the promoter region of the DRD4 gene, which is a promising target of future association studies. This sequence variation has been discovered during the large-scale application of our recently published *Sau*96 I RFLP genotyping method of the -616C>G SNP [30]. A novel technique was developed for the screening of this mutation, and applied on a Caucasian (Hungarian) population of 959 individuals.

## Results

**Figure 1:**
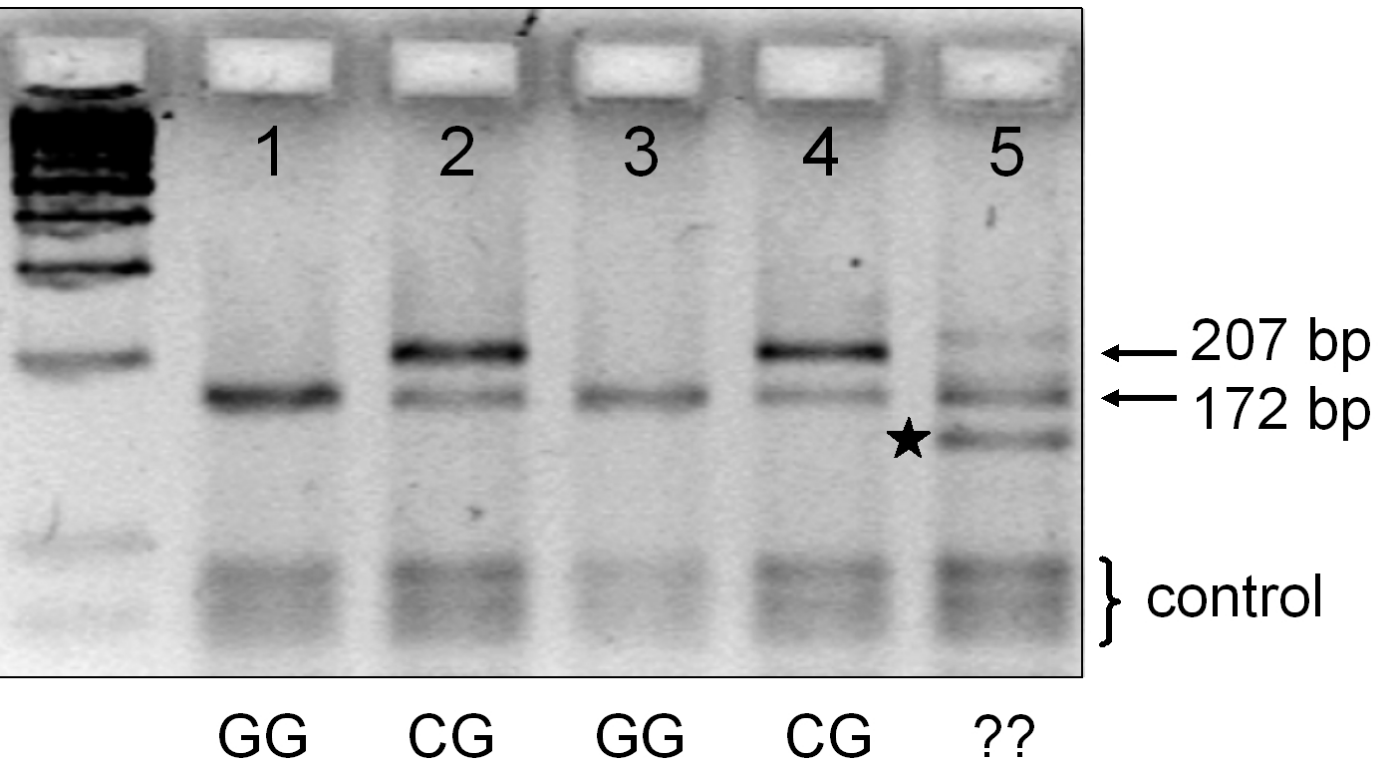
***Sau*96 I RFLP genotyping method of the -616C>G SNP reveals a new variation in the DRD4 gene. **Representative genotyping results are depicted. Conditions of PCR amplification and electrophoretic separation were carried out as previously described (see Figure 2. in [30]). The asterisk indicates the short PCR fragment containing the deletion.

Recently, we described a novel genotyping method for the analysis of the -616C>G SNP in the DRD4 gene [GenBank:L12397] promoter region [GenBank:U95122], consisting of the *Sau*96 I RFLP and an allele specific amplification procedure. The parallel application of the two independent methods (referred to as double genotyping) highly increases the reliability of the study, especially when investigating outstandingly polymorphic regions such as the DRD4 gene [30]. During the large-scale genotyping of the -616C>G SNP by this newly described double-genotyping method, we noticed some abnormalities in the size of the produced fragments. Fig. (1) unmistakably shows the two different size fragments obtained from the *Sau*96 I RFLP where the 207 bp long fragment represents the -616 C allele and the 172 bp long product refers to the cleaved -616 G allele. Surprisingly, in lane 5, an unexpected short fragment was produced (indicated by an asterisk) in case of a sample having a -616 GG genotype determined by allele-specific PCR [30] (not shown). The size of the indefinite fragment was approximately 30 bp less than that of the expected 172 bp long PCR product of the -616 G allele. The appearance of the unusual PCR fragment has two feasible explanations: (1) There might be a new SNP in the amplified fragments of the sample resulting in an additional polymorphic *Sau*96 I restriction site, (2) or a length variation in one of the alleles of the individual might also be responsible for the production of the short fragment. Electrophoretic analysis of the undigested PCR-products yielded two distinct bands demonstrating that the new sequence variant was a deletion.

When investigating the sample containing the deletion by allele-specific amplification no abnormalities were found (not shown). This can be readily explained focusing on the setup of this method, which applies a sense C-specific and an antisense G-specific primer for the simultaneous amplification of two PCR-products with different length corresponding to the two alleles respectively. Consequently, if the new sequence variant is localized upstream from the -616C>G SNP on the same chromosome as the -616 C allele or downstream from the SNP together with the G allele, then the deleted region is not amplified thus the mutation does not show up on the electropherogram. To confirm our notion we carried out the allele-specific PCR of this sample applying primers with opposite orientation (i.e. a sense G-and an antisense C-specific primer). In this arrangement we detected a shortened -616 G specific PCR product, suggesting that the deletion is localized downstream from the -616C>G SNP composing a haplotype with -616 G variant in this sample. Moreover, it is important to note that the deletion does not cause any uncertainty if the genotyping of the -616C>G SNP is carried out by allele specific amplification, as the size difference between the two allele-specific products is fairly big (252 bp) excluding the possibility of misgenotyping.

**Figure 2:**
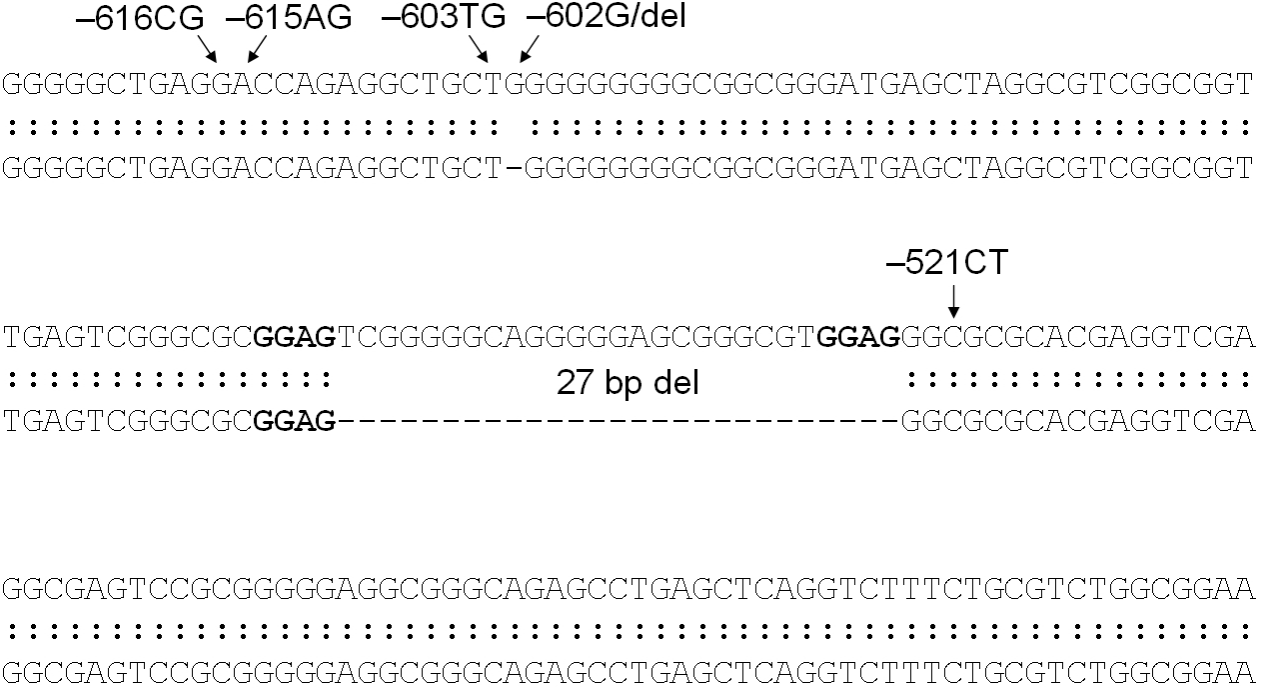
**Alignment of the deleted allele string with the published DNA sequence of the upstream region [GenBank:U95122, GenBank:L12397.] **Positions of the -616C>G, -615A>G, -603T>G, -602G>del and -521C>T SNPs are specified. The bold letters indicate the ambiguous localization of the deletion, however according to the nomenclature of the Human Genome Variation Society the suggested start point is the -524 position.

Sequencing of the shortened fragment of the *Sau*96 I RFLP (referred to as the product of the "Del" allele) also confirmed the presence of the deletion in this sample. Fig. (2) shows a sequence containing the deletion aligned to that obtained from the GeneBank : "Human dopamine D4 receptor gene, 5' flanking region" (accession number: U95122) and "Homo sapiens Dopamine D4 receptor (DRD4) gene" (accession number: L12397). It is unambiguous that the deletion affects 27 base-pairs, however its position cannot be marked explicitly since it is located between two GGAG sequences and it is hard to agree on which GGAG is enclosed by the deleted section. Therefore the mutation can be placed anywhere between the -524^th ^and the -554^th ^positions relative to the initiation codon. According to the nomenclature of the Human Genome Variation Society  we suggest the -524 position to be the assigned start point of the deletion, since this is the closest point to the coding sequence of the DRD4 gene.

**Figure 3:**
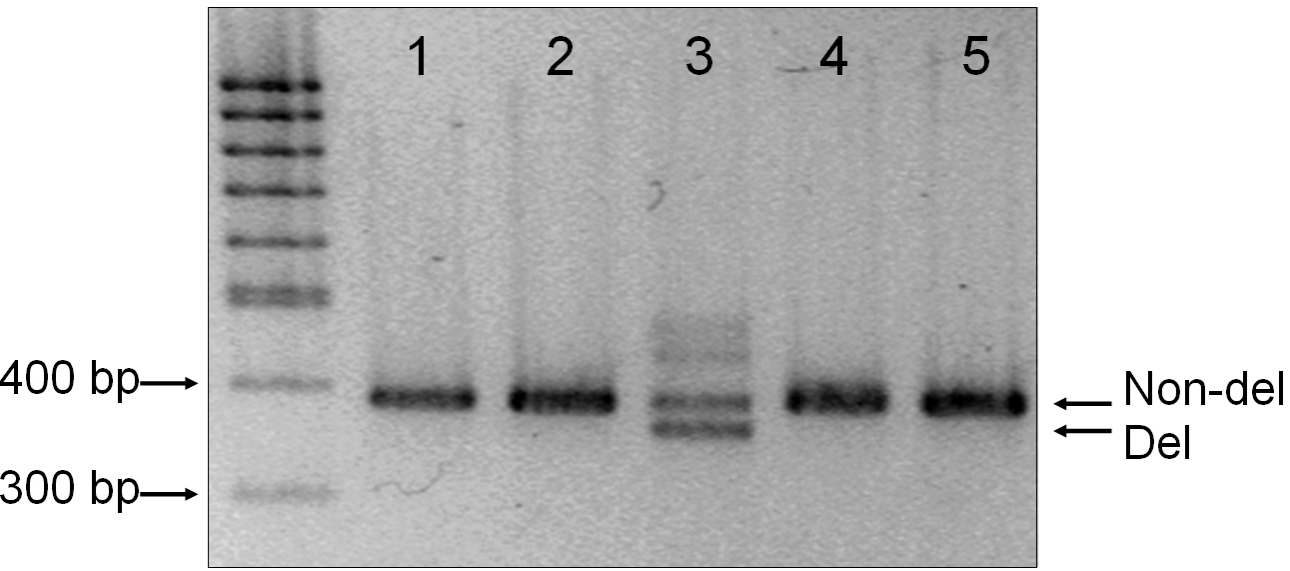
**Genotyping the 27 bp deletion mutation in healthy Hungarian individuals. **Amplification conditions: reaction mixtures contained 200 μM dATP, dCTP, dTTP, and 100 μM dGTP and dITP; 1 μM of each primer (see Methods), 1 ng DNA template, 0.25 U DNA polymerase, 1x reaction buffer, and 1x Q solution in a total volume of 10 μL. Thermocycling conditions: 95°C for 15 min, followed by 35 cycles of 94°C for 1 min; 65°C for 30 sec; 72°C for 1 min, finished by 72°C for 10 min. Electrophoresis: fine resolution agarose gel in 40 mM Tris, 10 mM EDTA pH 8.0 buffer solution at room temperature, 6.6 V/cm electric field, 90 min, followed by 1 μg/mL ethidium bromide staining. The faint diffuse bands in lane 3 migrating slower than the representative fragments presumably contain heteroduplex molecules.

PCR primers flanking the region of the mutation were designed (see "Methods") to determine the genotype of the 27 bp deletion in our population of unrelated Caucasians. Fig. (3) shows the electrophoretic separation of the obtained PCR amplicons in the analysis of 5 samples. A longer 391 bp (designated "Non-del") and a shorter 364 bp (referred to as "Del") fragment was formed in lane 3 representing a heterozygote for the 27 bp deletion ("Del"/"Non-del"). The rest of the individuals (lanes 1, 2, 4 and 5) do not contain the deletion: only the longer fragment was formed showing a homozygote "Non-del"/"Non-del" genotype. In order to determine the allele frequency of this new sequence variation, we screened for the Del allele in a large (*N *= 955) healthy, unrelated Hungarian (Caucasian) population. Seeing that our results show a very low allele frequency of 0.16% for the Del allele of the DRD4 promoter region, this new variant can be considered as a mutation rather than a polymorphism. The Mendelian inheritance of the novel 27 bp deletion was also investigated by a three-generation family analysis, where one heterozygote was found in the first and one in the second generation, respectively. We did not find any homozygotes in our sample population, or among the available relatives (*N *= 4) of the heterozygote individuals.

To assign the functional role of the 27 bp deletion we carried out an "*in silico*" transcription factor binding analysis. The Transcription Element Search System (TESS; ) was used to specify the transcription factors that can play a role in the gene expression regulation by binding to this region. Even when applying stringent search parameters (6 bases as "Minimum string length" and allowing no mismatch in the sequence) the following human transcription factors were suggested to bind the region of investigation: NF-E2, AP-2, Sp1. Although the physiological importance of this result needs to be further studied, the presence of the Sp1 site is especially notable, as a zinc finger type transcription factor (dopamine receptor regulating factor, DRRF) was described to effectively bind to the same sequences as Sp1 in the dopamine receptor promoters [48].

## Discussion

Here we described a novel 27 bp deletion in the DRD4 promoter region, which has several important aspects. Primarily, it is a source of misgenotyping of the -616C>G SNP when using the standard genotyping method of *Sau*96 I RFLP. As this SNP is often studied in psychiatric genetics finding the appropriate genotyping methodology is of great importance [39,40]. The problem arises from the hardly detectable difference between the 180 bp amplicon of the 27 bp Del and the -616 C allele-combination (i.e. haplotype) vs. the 172 bp product of the -616 G variant after *Sau*96 I digestion. Thus, the existence of such a deletion is a source of error, since some of the -616 C alleles might be misgenotyped as -616 G in the presence of the deletion.

On the other hand, using the previously reported [30] allele-specific amplification, it is possible to distinguish between the -616C and G alleles with no doubt, even in the presence of the deletion. We can explain the results assuming that the four samples heterozygous for the deletion contained the deleted allele on the same chromosome as the -616G allele. This assumption was later confirmed by allele specific sequencing. As the -616C-specific primer was the sense and the -616G-specific primer was the antisense primer in our system, the obtained products did not contain the region of the deletion. When applying a -616 G-specific sense primer in another setup of allele specific amplification, the G-specific fragment clearly showed the presence of the 27 bp deletion (data not shown), but the length variation did not influence the accuracy of the -616C>G SNP genotyping after all.

Although the occurrence of the 27 bp deletion is rather low (0.16%) in our healthy Caucasian population, the allele frequency might be different in other ethnic groups and various clinical samples. Therefore, further studies are necessary to gather information on the allele and genotype frequencies of this novel length variant in a range of other populations, and these results should be considered in the psychogenetic association studies of the -616C>G polymorphism as well.

It is of additional importance to perform association studies in different populations since each may represent distinct environments that could possibly interact with genetic variants. The methodological conditions and demographic setting is crucial in evaluating the strength of a relationship and in replicating an association found by different investigations. Here we address the differences in ethnic structure and methodological approach used in the successive studies of commonly investigated DRD4 gene variations and review the diversity of their allele frequencies in different populations around the globe.

The variable number of a 48 bp repeat (48 bp VNTR) in exon III makes the DRD4 gene one of the most frequently studied genes in psychiatric genetics. The "long form" (7R) of this length polymorphism was found to be associated with the personality trait of "novelty seeking" [12,13], children's attention deficit hyperactivity syndrome [8] and drug abuse [10]. In the Hungarian population (as well as in other Caucasian samples) the 4 repeat allele occurs most frequently (65.0%), followed by the 7 repeat (19.5%) and the 2 repeat (8,9%) variations. The rare alleles include the 3 repeat (3.8%), 8 repeat (1.2%), 5 repeat (1.1%) and the 6 repeat (0.4%) variants, whereas no 9 and 11 repeat allele was found (see Table 1). We have identified one 10 repeat allele (0.1%) in our large (2N = 1196) Caucasian sample. This form has only been reported previously in the African population with 1% incidence [49]. In numerous studies, genotype groups were defined only by absence vs presence of the DRD4 exon III 7 repeat, these results are not included in the table.

**Table 1:** Allele frequency data for the 48 bp VNTR polymorphism of DRD4

**Origin of populations**		**Allele frequency (%)**								
		
		**2N**	**2**	**3**	**4**	**5**	**6**	**7**	**8**	**10**
**Caucasian**	Hungarian (Ronai et al., 2000)	1196	8.9	3.8	65.0	1.1	0.4	19.5	1.2	0.1
	European-mixed (Chang et al., 1996)	176	12.0	6.0	57.0	2.0	1.0	21.0	1.0	0.0
	Caucasians in New Zealand (Mill et al., 2002)	1760	8.8	4.6	65.0	0.9	0.6	19.4	0.6	0.0
	German (Strobel et al., 2003)	230	7.3	4.3	66.5	0.9	0.4	19.1	1.3	0.0
	German (Franke et al., 2000)	394	7.1	3.3	70.1	3.3	0.5	15.2	0.5	0.0
	Italian (Mochi et al., 2003)	212	10.4	3.3	68.9	2.8	0.9	13.2	0.5	0.0
	Italian (DeLuca et al., 2003)	190	11.0	5.3	67.9	0.5	0.5	13.7	1.0	0.0

	*Mean*		**8.9**	**4.3**	**65.6**	**1.3**	**0.5**	**18.5**	**0.8**	**0.0**
	*Standard deviation*		1.1	0.6	2.5	0.8	0.2	2.1	0.3	0.0

**Asian**	Chinese (Li et al., 2000)	608	18.8	1.2	76.6	1.3	2.1	0.0	0.0	0.0
	Japanese (Ishiguro et al., 2000)	680	12.0	1.0	81.0	4.0	1.0	1.0	0.0	0.0

	*Mean*		**15.2**	**1.1**	**78.9**	**2.7**	**1.5**	**0.5**	**0.0**	**0.0**
	*Standard deviation*		3.4	0.1	2.2	1.3	0.5	0.5	0.0	0.0

**African**	Falashan (Chang et al., 1996)	128	3.0	0.0	83.0	0.0	2.0	11.0	0.0	1.0

**American**	Mayans (Chang et al., 1996)	100	1.0	0.0	57.0	0.0	3.0	39.0	0.0	0.0

The prevalence of the most common alleles in the Hungarian population studied matches the frequencies measured by Mill et al. (New Zeland [50]) and Strobel et al. (Germany [51]). Minor differences of the 7 repeat frequencies incidence among Caucasians might originate from population stratification. If, however, allele frequencies of similar populations are quite different this problem might also originate from preferential amplification of shorter PCR products [52] as a methodological artifact in this highly GC rich region, resulting in an underestimation of the 7 repeat allele frequencies in the above populations.

Noticeably, there is considerable variation in the distribution of the alleles as we compare Caucasian and Asian populations. The occurrence of the 7 repeat allele is extremely low (0.5%) in Chinese and Japanese samples, and relatively high in North American Mayans (39%), whereas Caucasians have an intermediate (18.5%) mean prevalence. There are two theories describing the origin of the 7 repeat allele [53,54], however independently on the reason for the varying frequency data of this variation, a high danger of artifacts caused by population admixture should definitely be taken into account when genetic association studies are being performed [55].

Beside the length polymorphism of the DRD4 gene coding sequence, the 5' upstream region of this gene has numerous sequence variations also. Among the promoter polymorphisms, the -521C>T SNP has been widely studied since it was shown to be associated with the personality trait of novelty seeking [16,34]. Frequency data obtained for the -521C>T SNP seem to be considerably constant among the populations (see Tab. 2), as frequency differences between the ethnic groups are smaller than the variations observed within the same study group. Therefore, the alterations in the frequency between the C vs. T alleles can be readily explained by the differences in methodology and sample size. One of the possible sources of technical difficulties might originate from using the PCR-RFLP protocol designed by Okuyama et al. [32], where the 3' end of the reverse primer anneals to the polymorphic -603^rd ^position [41], -603T>del), which might result in unequal amplification of the homologous chromosomes in heterozygotes. Therefore the position of the primers were changed in other protocols, including the previously described PCR-RFLP system, where an internal control restriction site was added to the system [16]. In order to enhance the validity and reliability, two parallel methods were used for genotyping the -521C>T SNP in our laboratory, a PCR-RFLP and a fast allele-specific protocol [56]. Using a large sample (N = 598), we found a somewhat lower (46.5%) allele frequency for the -521 C allele compared to the -521 T allele and the genotype frequencies corresponded to the Hardy-Weinberg equilibrium (see Table 2., line 1).

**Table 2:** Genotypes and allele frequencies of the -521 CT SNP in different populations

**Origin of population**		**Allele frequency (%)**			**Genotype frequency (%)**			
		
		**2N**	**C**	**T**	**N**	**CC**	**CT**	**TT**
**Caucasian**	Hungarian (Szantai et al., 2005)	1196	46.5	53.5	598	21.4	50.0	28.6
	European-mixed (Barr et al., 2001)	308	45.8	54.2	154			
	German (Strobel et al., 2003)	230	40.0	60.0	115	20.9	38.3	40.8
	Swedish (Jönsson et al., 2001)	776	42.0	58.0	388	15.0	54.0	31.0

	*mean*		**44.4**	**55.6**		**19.1**	**50.2**	**30.7**
	*Standard deviation*		2.4	2.4		3.0	4.5	3.6

**Asian**	Japanese (Okuyama et al., 1999)	538	41.0	59.0	269	14.0	53.0	33.0
	Japanese (Ishiguro et al., 2000)	538	41.0	60.0	269	14.0	53.0	33.0
	Japanese (Mitsuyasu et al., 1999)	294	41.0	59.0	147	17.9	46.3	35.8
	Japanese (Okuyama et al., 2000)	172	53.5	46.5	86	32.5	41.9	25.6
	Chinese (Xing et al., 2003)	412	39.1	60.9	206	12.1	53.9	34.0
	Chinese (Li et al., 2000)	422	40.3	59.7	211	27.5	25.6	46.9
	Korean (Lee et al., 2003)	202	46.0	54.0	101			

	*mean*		**41.8**	**58.4**		**17.9**	**46.7**	**35.5**
	*Standard deviation*		3.5	3.6		6.7	10.4	5.8

**American**	Afro-American (Bookman et al., 2002)	142	55.0	45.0	71	32.0	42.0	26.0

Although the literature of the -616C>G SNP is limited, the available genotype frequencies do vary among different ethnic groups significantly as shown in Tab. (3). The frequency of the two alleles were quite similar (-616C: 51.5%, -616G: 48.5%) in the Hungarian population studied, whereas in Asian and Afro-American populations a higher occurrence of the -616G allele (69.7% and 71.8% respectively) was observed. It is difficult to account for the significant difference between the mixed-European and the Hungarian samples. According to our recent results [30], the *Ava *II RFLP genotyping protocol [29] overestimated the -616C allele in the presence of the -615G allele of a novel SNP in this position. *Sau*96 I, in place of the *Ava *II restriction enzyme, was shown to be the appropriate choice of restriction endonuclease [30], as the newly described -615A>G SNP had no influence on the digestion reaction by *Sau*96 I. This is another example of how obtained allele frequencies depend on differences in methodology, providing a source of unreplicated association studies.

**Table 3:** Allele and genotype frequencies of the -616 CG SNP in 5 populations

**Origin of population**		**Allele frequency (%)**			**Genotype frequency (%)**			
		
		**2N**	**C**	**G**	**N**	**CC**	**CG**	**GG**
**Caucasian**	Hungarian (Szantai et al., 2005)	1196	51.5	48.5	598	27.8	47.5	24.7
	European-mixed (Barr et al., 2001)	308	72.7	27.3	154			

	*mean*		**55.8**	**44.2**		31.1*	49.3*	19.5*
	*Standard deviation*		8.6	8.6				

**Asian**	Japanese (Mitsuyasu et al., 1999)	160	28.4	71.6	80	14.9	26.9	58.2
	Chinese (Xing et al., 2003)	412	31.1	68.9	206	6.3	49.5	44.2

	*mean*		**30.3**	**69.7**		**8.7**	**43.2**	**48.1**
	*Standard deviation*		1.2	1.2		3.9	10.1	6.3

**American**	Afro-American (Bookman et al., 2002)	142	28.2	71.8	71	13.0	31.0	56.0

* Expected genotype frequencies were calculated from measured allele frequencies assuming Hardy-Weinberg equilibrium in the populations.

Recently, significant amount of interest has been focused on a polymorphic tandem repeat element located 1.2 kb upstream of the initiation codon in the DRD4 promoter. The 120 bp of duplicated sequence was linked to ADHD and might be involved in the regulation of transcription [43,57]. Allele frequency variation is high among populations, in Europeans the duplicated form is more common than in other populations (see Tab. 4). Since the region of this length variation is not GC rich and it is further away from the polymorphic hotspot of the DRD4 promoter, no significant genotyping obstacles have been found.

**Table 4:** Frequency distribution for the 120 bp duplicated alleles of the DRD4 promoter

**Origin of population**		**Allele frequency (%)**			**Genotype frequency (%)**			
		
		**2N**	**1**	**2**	**N**	**1/1**	**1/2**	**2/2**
**Caucasian**	Hungarian (Szantai et al., 2005)	1196	17.1	82.9	598	3.0	28.3	68.7
	European-mixed (Seaman et al., 1999)	174	19.5	80.5				
	European-mixed (Barr et al., 2001)	308	19.9	80.1				

	*mean*		**17.9**	**82.1**		3.2*	29.4*	67.4*
	*Standard deviation*		1.5	1.5				

**Asian**	Chinese (Xing et al., 2003)	412	37.4	62.6	206	13.6	47.6	38.8
	Chinese (Seaman et al., 1999)	122	36.1	63.9				

	*mean*		**37.1**	**62.9**		13.8*	46.6*	39.6*
	*Standard deviation*		0.5	0.5				

**African**	African (Seaman et al., 1999)	136	59.6	40.4		35.5*	48.2*	16.3*

**American**	Mayan (Seaman et al., 1999)	106	48.1	51.9		23.1*	49.9*	26.9*

* Expected genotype frequencies were calculated from measured allele frequencies assuming Hardy-Weinberg equilibrium in the populations.

Fig. (4) summarizes the polymorphisms highlighted by the literature, and the number of the variants keeps increasing. Some of the recently described polymorphisms such as the -1106C>T, -906C>T [27], -615A>G [30] SNPs and the novel 27 bp deletion, described here have not been characterized yet more thoroughly. The 27 bp deletion can be rapidly genotyped with the assay described in this paper and could be a component of further association studies that analyze the 5' region of the DRD4 gene. Additionally, the dbSNP database of the NCBI contains numerous further SNPs in the non-coding region of the gene (-872A>G, -844C>G, -764A>C, -754C>G, -713C>G, -599C>G, -528C>T, -364A>G) that haven't even been published yet. Theoretical approaches have shown that highly variable polymorphic markers are more useful in association analysis than less polymorphic ones, since there is a higher probability to identify allele frequency differences between cases and controls [58]. Similarly, analysis of haplotypes, involving several polymorphic sites, might provide a greater power for association analysis. Haplotypes of the 120 bp duplication [31] and some of the SNPs [23,29] published earlier have already been the targets of numerous genetic association studies. Recently, direct haplotype detection methods for the commonly investigated -521C>T and -616C>G SNPs as well as the 120 bp duplication promoter polymorphisms were developed in our laboratory [59,60]. Linkage disequilibrium analysis of polymorphisms marks the different haplotype blocks on the chromosome segment giving way to future association studies [61].

The polymorphic variations in the regulatory region may directly influence the regulation of transcription of the DRD4 gene. It was demonstrated that some of these polymorphisms have functional effects resulting in different transcriptional activity [32,57]. Furthermore, the 120 bp duplication and the C to G change at the -616^th ^position cause the gain of additional binding sites of known transcription elements [31,39,62]. Accordingly, we examined the deleted sequence for any potential transcriptional binding sites and found the consensus sequences of several factors, including *Sp1*, *AP-2alphaB *and *NF-E2*. It has been reported in a human retinoblastoma cell line that the -521C>T SNP has significant influence on the transcriptional efficiency of the DRD4 gene suggesting the relevance of a single SNP in dopaminergic neurotransmission [32]. As the 27 bp deletion lies in the very same region, it can be assumed that this variation might also have a considerable impact on the transcriptional activity of the gene.

## Methods

### Participants

DNA was extracted from epithelial cells of 959 healthy subjects (396 male and 563 female) in a Caucasian sample of Hungarian origin. Signed informed consent was obtained from all the participants. The research protocol was approved by the Research Ethics Committee.

### Non-invasive DNA sampling

Buccal cells were collected by cotton swabs from the inner surface of the mouth [63]. DNA was isolated by phenol extraction and alcohol precipitation as described earlier [64].

### Genotyping protocol for the 27 bp del

The Qiagen^® ^HotStarTaq™ DNA polymerase kit was used for polymerase chain reaction (PCR). Reaction mixtures contained 200 μM dATP, dCTP, dTTP, and 100 μM dGTP and dITP; 1 μM of forward primer (5'-GGA ATG GAG GAG GGA GCG GG-3'), and 1 μM of reverse primer (5'-GAC GCC AGC GCC ATC CTA CC-3'), approximately 1 ng DNA template, 0.25 U DNA polymerase, 1x reaction buffer, and 1x Q solution in a total volume of 10 μL. Thermocycling was initiated at 95°C for 15 minutes to activate the hot start enzyme and to denature genomic DNA, which was followed by 35 cycles of 1 min denaturation at 94°C, 30 sec annealing at 65°C and 1 min extension at 72°C. A final 10 min extension step at 72°C was followed by cooling the samples to 8°C. PCR products were analyzed by conventional submarine horizontal agarose gel-electrophoresis (MidiGel of Biocenter, Szeged, Hungary). A composite gel was applied containing 1.5% low EEO agarose Type I (Sigma Chemicals) and 2% Methaphor agarose "fine resolution" (Cambrex Corporation). Separation was performed at room temperature in 40 mM Tris, 10 mM EDTA.Na_2_, 1% acetic acid (pH 8.0) buffer and 6.6 V/cm (100 V field strength, 26 mA) for 90 min. After separation, dsDNA fragments were stained in 1 μg/mL ethidium bromide solution at room temperature for 15 minutes. No destaining was necessary. A BioRad Gel-Doc 1000 gel-documentation system (Hercules) was used for visualization of the DNA fragments.

### DNA sequencing

Genomic DNA of individuals shown to carry the deleted allele were sequenced by amplification of the region in question using the above described PCR conditions. Polymerase chain reaction was carried out by the forward primer: 5'-GGA ATG GAG GAG GGA GCG GG-3', and reverse primer: 5'-CGC TCC ACC GTG AGC CCA GTA T-3'. The shorter PCR product (deleted allele) was purified from the gel using the Qiagen QIAquick Spin DNA-Extraction Kit. DNA sequencing was performed by an ABI 370 Sequencer, using the reverse primer (see above) of the amplification reaction.

### Database searching

The 27 bp of sequence, which is deleted in the mutant allele, was searched for known transcription factor binding sites using the Transcription Element Search Software (TESS) web-based search tool to search the TransFac database; [65].

## Authors' contributions

ES carried out the genotyping experiments of the 959 individuals, took part in the elaboration of the novel genotyping procedure and helped to draft the manuscript. RS analyzed the results of the initial experiments suggesting the presence of the novel mutation and drafted the manuscript. MS conceived of the study and participated in its design and coordination. AG carried out the database search and coordinated the experimental work. ZR designed the primers for the investigation of the 27 bp del and optimized the genotyping protocol, analyzed the sequencing results and helped to finalize the manuscript. All authors read and approved the final manuscript.
